# Diltiazem is a useful and effective medication for reversal of coronary artery spasm-induced complete atrioventricular block: A case report

**DOI:** 10.3389/fcvm.2023.1134658

**Published:** 2023-04-03

**Authors:** Jin Zhang, Li Liu, Chengwei Liu, Min Han, Chengyi Xu, Rujie Qiu

**Affiliations:** ^1^ Division of Cardiac Care Unit, Department of Cardiology, Wuhan Asia Heart Hospital, Wuhan, China; ^2^ Department of Cardiology, Medical College of Wuhan University of Science and Technology, Wuhan, China

**Keywords:** coronary artery spasm, fatal arrhythmia, complete atrioventricular block, calcium channel blocker, diltiazem

## Abstract

Coronary artery spasm (CAS) is characterized by reversible diffuse or focal vasoconstriction, a phenomenon that plays an important role in the pathogenesis of ischemic heart disease. Fatal arrhythmias, such as ventricular tachycardia/fibrillation and complete atrioventricular block (AV-B), are very common in patients with CAS. Nondihydropyridine calcium channel blockers (CCBs) such as diltiazem were recommended as first-line medications for treating and preventing CAS episodes. However, its use remains controversial in CAS patients with AV-B as this type of CCB can also cause AV-B itself. Here, we present the use of diltiazem in a patient with complete AV-B caused by CAS. The patient's chest pain was rapidly relieved, and complete AV-B was promptly restored to sinus rhythm following the administration of intravenous diltiazem without any adverse effects. In this report, we highlight the useful and effective application of diltiazem for treating and preventing complete AV-B caused by CAS.

## 1. Introduction

Coronary artery spasm (CAS) is defined as a severe reversible diffuse or focal vasoconstriction, a phenomenon that plays an important role in the pathogenesis of ischemic heart disease (1). Unlike classical angina, which is induced by emotional or physical stress, CAS usually occurs at rest or during regular daily activity. The clinical manifestations of CAS include variant angina, acute myocardial infarction (AMI), fatal arrhythmias [e.g., ventricular tachycardia/fibrillation (VT/VF), complete atrioventricular block (AV-B)], and even sudden cardiac death (2, 3). Vasodilators such as nitrates and calcium channel blockers (CCBs) are considered effective first-line treatment for the prevention of vasoconstriction episodes; however, the use of CCBs in patients with CAS-induced complete AV-B remains controversial as CCBs can also reduce cardiac output and cause AV-B themselves (4–6). Here, we present a case of complete AV-B with hypotension caused by CAS, where the patient's severe chest pain was relieved and complete AV-B was restored back to sinus rhythm following the administration of intravenous diltiazem without any observed adverse effects. This patient was subsequently prescribed diltiazem long-term for the prevention of CAS. In this case report, we aim to highlight the useful and effective application of this medication for treating and preventing CAS-induced complete AV-B.

## 2. Case presentation

**Figure 1:**
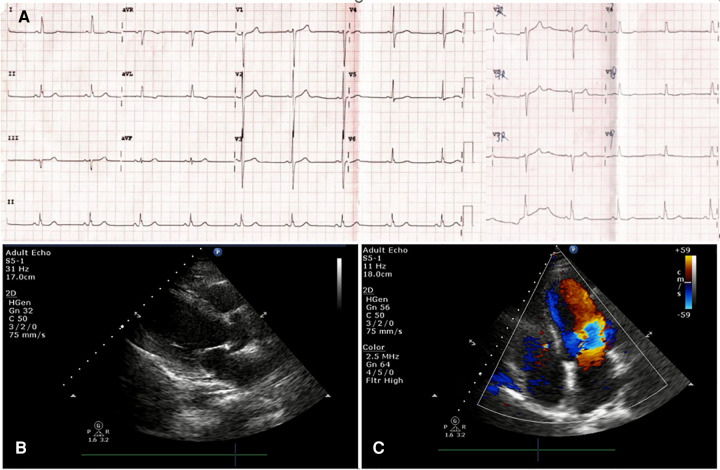
Emergency electrocardiogram (ECG) was performed at rest showed normal sinus rhythm with no significant ST-T changes in all leads (**A**); Echocardiography in ER indicated a mildly thickened ventricular septum and left ventricular posterior wall, mild tricuspid regurgitation and a slightly reduced left ventricular ejection fraction (LVEF 50%) (**B** and **C**).

**Figure 2:**
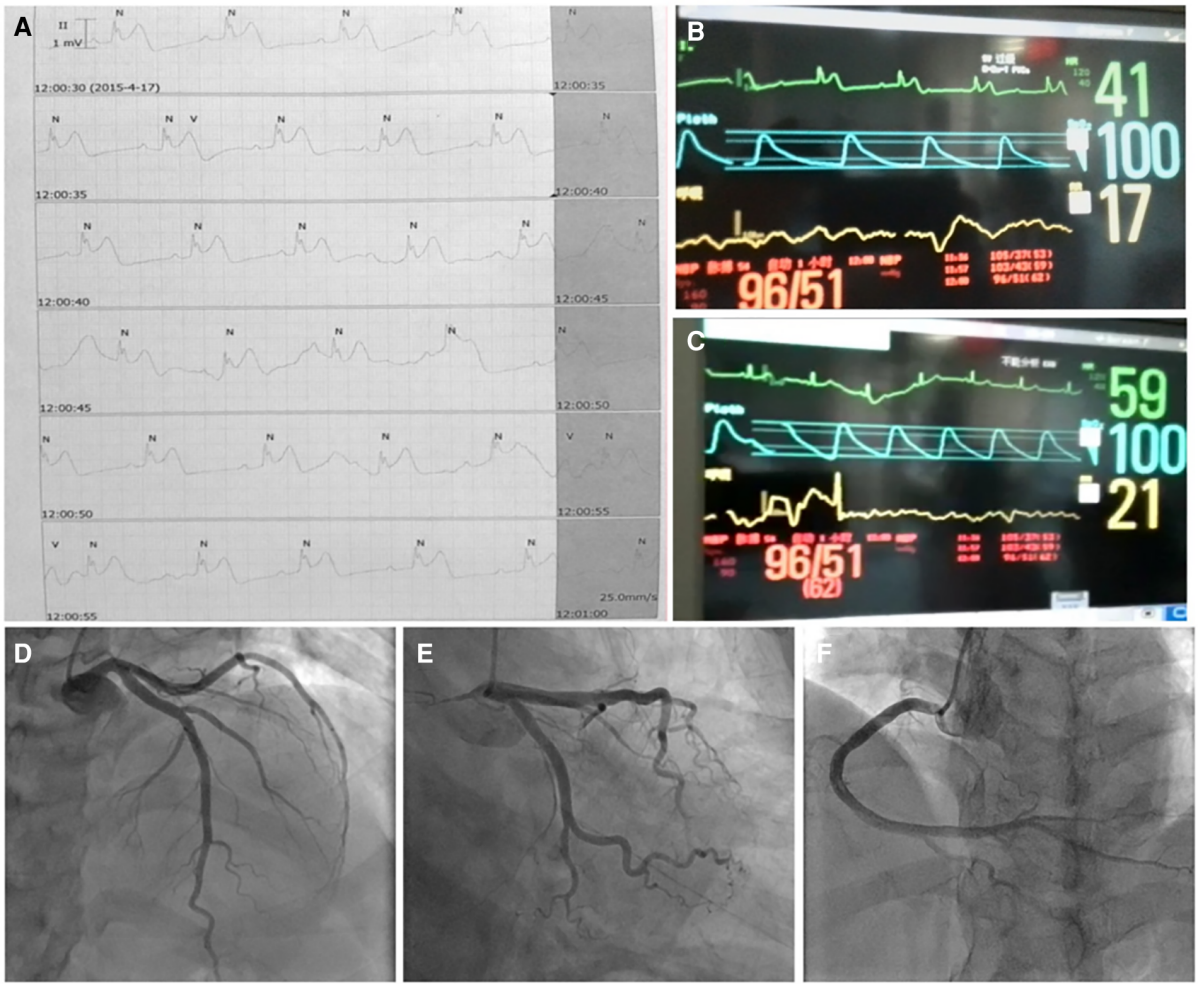
Twelve minutes after hospitalization, the chest pain recurred, and monitoring ECG revealed ST-T segments elevation in lead II and complete AV-B with HR 41 bpm accompanied by hypotension (BP 96/51 mmHg) (**A,B**). Five milligrams of diltiazem was administered intravenously with prompt relief of chest pain, and complete AV-B was converted back to sinus rhythm (**C**). Coronary angiography on day 2 revealed nonobstructive coronary arteries in the LAD, (**D**), LCX, or (**E**) RCA (**F**). ECG, electrocardiogram; AV-B, atrioventricular block; HR, heart rate; LAD, left anterior descending artery; LCS, left circumflex branch; RCA, right coronary artery.

**Figure 3:**
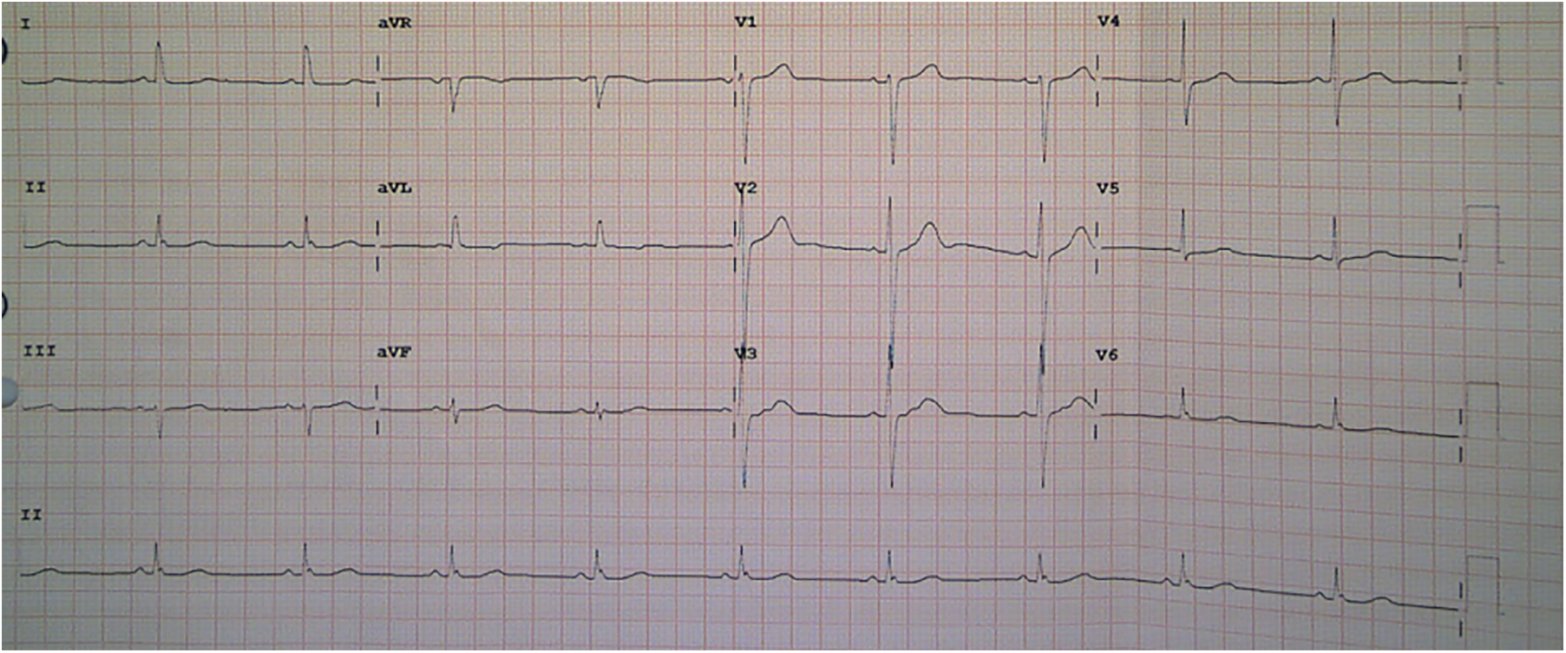
Repeated ECG revealed normal heart rate without ST-T changes after diltiazem treatment. ECG, electrocardiogram.

A 44-year-old female patient presented to the emergency room (ER) of our hospital with complaints of “intermittent chest pain and chest tightness for two weeks, worsening for the past 2 h.” She reported that the chest pain that started 2 weeks before she attended the ER involved intermittent retrosternal pain occurring at midnight at rest, accompanied by tightness in her throat without shortness of breath, palpitations, or diaphoresis. The symptoms lasted for a few minutes and were relieved spontaneously. Her symptoms were recurring and became increasingly more frequent. Her medical history included hypertension for 7 years and noncompliance with antihypertensives (irbesartan, 150 mg/day). She also reported abnormal glucose tolerance for the past year. She denied taking any other medicines. Physical examination upon admission revealed that vitals were all within normal limits: blood pressure 105/51 mmHg (1 mm Hg = 0.133 kPa), heart rate (HR) 56 beats/min, respiratory rate 20 /min, and oxygen saturation level at 99% (room air). Cardiac and pulmonary examination revealed normal heart sounds without murmur and clear lungs to auscultation. The abdomen was soft and nontender to palpation, without rebound tenderness. There was no appreciable bilateral lower extremity edema. The electrocardiogram (ECG) performed at rest showed normal sinus rhythm with no significant ST-T changes (Figure 1A); echocardiography completed in the ER showed a mildly thickened ventricular septum and left ventricular posterior wall, mild tricuspid regurgitation, and slightly reduced left ventricular ejection fraction (LVEF 50%, normal range: 55%–75%) (Figures 1B,C). ER laboratory tests indicated normal troponin I of 0.016 ng/mL (normal range, 0–0.04 ng/mL), creatine kinase MB isoenzyme of 0.7 ng/mL (normal range, 0–5.0 ng/mL), and myoglobin of 14.1 ng/mL (normal range, 0–70.0 ng/mL) levels. Since acute coronary syndrome (ACS) cannot be excluded, a loading dose of aspirin (300 mg) + clopidogrel (300 mg) was prescribed and coronary angiography (CAG) was suggested but was refused by the patient and her family members. The patient was then admitted to the cardiac care unit (CCU) for suspicion of ACS. Twelve minutes after hospitalization, her chest pain recurred, and monitoring ECG revealed ST-segment elevation in lead II and complete AV-B with HR at 41 beats/min accompanied by hypotension (BP at 96/51 mmHg) (Figures 2A,B). According to the characteristics of the patient's chest pain, ECG (near normal when chest pain was relieved), and biomarker (normal troponin I) obtained in ER, the diagnosis of CAS was highly suspected. With a possible diagnosis of CAS, 5 mg of diltiazem was administered intravenously with prompt relief of chest pain, and complete AV-B was converted back to sinus rhythm (HR at 59 beats/min) (Figure 2C). The patient was on 50 mg of diltiazem hydrochloride intravenously (at 5 mL/h) for maintenance. She received CAG the second day after a repeated suggestion by the physician, which revealed normal left main branch, 20%–30% stenosis at the opening and middle segment of the left anterior descending artery (LAD), 30% stenosis at the first diagonal branch (D1), 30% stenosis at the beginning of the circumflex branch (LCX), and 20% stenosis at the middle of the right coronary artery (RCA) (Figures 2D,E,F, Supplementary Videos S1–S3). Routine lab work during hospitalization revealed that white blood cell count, lipids, liver function, renal function, glycosylated hemoglobin, electrolytes, thyroid function, coagulation function, erythrocyte sedimentation rate, D-dimers, cytokines, and urinalysis were all within normal limits. Based on the patient's symptoms, ECG, and CA findings, a diagnosis of CAS was made. Repeated troponin (0.031 ng/mL) and ECG were normal (Figure 3). The patient was then prescribed diltiazem sustained-release capsules (90 mg, twice per day), an antiplatelet medication (aspirin, 100 mg per day), and lipid-regulating medication (atorvastatin, 20 mg per day). With other in-patient treatments and improving myocardial ischemia, the patient no longer had chest pain or discomfort during hospitalization. The patient was followed up on an outpatient basis without recurrence of chest pain or other reported symptoms. At her last follow-up appointment, she had remained symptom-free for the past 3 months and was in good clinical condition. The timeline of this case report is provided in Table 1.

**Table 1:** Timeline of the case report.

Day 1, 11:33	Hospitalization
Day 1, 11:45	Chest pain recurred with complete AV-B
Day 1, 12:03	Diltiazem was intravenously
Day 1, 12:05	Chest pain revealed and complete AV-B to sinus rhythm
Day 2	CAG
Day 4	Discharge

AV-B, atrioventricular block; CAG, coronary angiography.

## 3. Discussion

CCBs are often classified into two major categories, either nondihydropyridines such as diltiazem and verapamil or dihydropyridines such as nifedipine, amlodipine, and felodipine. CCBs noncompetitively inhibit the influx of extracellular calcium ions across the myocardial and vascular smooth muscle cell membranes during depolarization by binding to the L-type voltage-gated calcium channels on the myocardium and vascular smooth muscle of the coronary arteries, weakening the contractility of the myocardium and causing coronary artery dilatation (7, 8). As a nondihydropyridine CCB, diltiazem also has inhibitory effects on atrioventricular (AV) conduction through its ability to impede slow calcium channel function, resulting in a reduced heart rate (8, 9). Diltiazem-induced AV-B was commonly seen in the clinical setting (3, 4, 9) but was thought to be reversible upon withdrawal of the medication.

CAS is one of the most common types of coronary vasomotor disorders. According to the International Study Group on Coronary Vasomotor Disorders published in 2017 (10), the diagnostic criteria for vasospastic angina include: (1) typical coronary spastic angina (e.g., resting chest pain occurring nocturnally or early morning) that is relieved by nitrates or CCB; (2) transient ischemic ECG changes, including ST-segment elevation or depression and new negative U waves; and (3) manifestations of coronary spasm, i.e., transient coronary artery spasm under acetylcholine, ergometrine, hyperventilation challenge tests or (4) spontaneous spasm with transient nonfixed stenosis of >90%, accompanied by angina pectoris and ECG ischemic changes. Based on ECG and CAG findings, CAS is classified as “definitive” or “suspected” vasospastic angina. Definitive vasospastic angina is diagnosed if nitrate-responsive angina is evident during spontaneous episodes and either the transient ischemic ECG changes during spontaneous episodes or coronary artery spasm criteria are fulfilled. Suspected vasospastic angina is diagnosed if nitrate-responsive angina is evident during spontaneous episodes, but transient ischemic ECG changes are either equivocal or unavailable and the coronary artery spasm criteria are equivocal. Although the underlying mechanisms behind CAS remain elusive, endothelial dysfunction, autonomic nervous system disorders, and hyperreactivity of vascular smooth muscle cells were thought to contribute to the phenomenon (1, 3). Normally, endothelial cells produce nitric oxide (NO), a potent vasodilator that functions as a suppressor for vasoconstrictive metabolites (11). Endothelial dysfunction results in a deficiency of endogenous NO; therefore, circulating vasoactive substances will favor vasoconstriction and underlie a nonspecific enhancement of the response to all vasoconstrictor stimuli (12). Several clinical studies have confirmed the impaired endothelial NO bioactivity in epicardial coronary arteries of patients with CAS (13, 14). Furthermore, endothelial dysfunction in a setting of normal coronary arteries proved by selective loss of acetylcholine-induced vasodilatation has been suggested as a sign of the future development of atherosclerosis (15). Prolonged vasospasm causes cardiac ischemia and therefore easily induces acute myocardial infarction, heart failure, sudden cardiac death, and fatal arrhythmias, such as VT/VF or complete AV-B (16). CCBs are currently recommended as the first-line treatment for CAS due to their effectiveness in the remission and prevention of CAS (3, 17). However, their use in CAS patients with hypotension and bradycardia was controversial because CCBs can reduce cardiac contractility and cause AV-B (4, 5). According to the drug use instructions, nondihydropyridine CCBs (e.g., diltiazem, verapamil) are contraindicated in patients with severe hypotension, cardiogenic shock, second- or third-degree AV block, sick sinus syndrome, persistent sinus bradycardia, and sinus arrest due to the possibility of causing bradycardia and worsening cardiac output. However, studies have shown (6, 18) that diltiazem was safe and effective in the treatment of AV-B caused by coronary spasm; therefore, it is not absolutely contraindicated in the management of malignant arrhythmia caused by CAS. In our patient, despite both complete AV-B and hypotension, 5 mg of intravenous diltiazem was administered immediately with significant improvement of symptoms after about 1 min, and ECG monitoring indicated restoration of sinus rhythm. The patient did not suffer any adverse effects from drug administration. We thought that the therapeutic effect was driven by the aggregate of several mechanisms of action of diltiazem, including a reduction in the contractile processes of the myocardial smooth muscle cells, vasodilation of the coronary and systemic arteries (including epicardial and subendocardial), and reduction in heart rate, resulting in lowering of myocardial oxygen demand. However, we must point out that an emergency CAG would have been appropriate if ACS is suspected in clinical settings. CCB is not routine management, only when CAS is highly suspected or confirmed and CA is unavailable or incompetent (e.g., unable to obtain written patient consent). CAG is still recommended even with symptom relief because coronary vasospasm may occur in the presence of fixed atherosclerotic obstructions in the epicardial coronary arteries (19).

## 4. Conclusion

In summary, this case report provided additional evidence that diltiazem is a useful and effective medication in managing CAS-induced angina and complete AV-B. It can achieve immediate efficacy during intravenous use; however, large studies are warranted to confirm the findings presented here.

## Data Availability

The original contributions presented in the study are included in the article/**Supplementary Material**, further inquiries can be directed to the corresponding authors.
